# The risk of type 2‐diabetes among persons with intellectual disability: a Danish population‐based matched cohort study

**DOI:** 10.1111/jir.13190

**Published:** 2024-10-02

**Authors:** A. Thorsted, S. F. Lehn, A. Kofoed‐Enevoldsen, A. Andersen, A. Heltberg, S. I. Michelsen, L. C. Thygesen

**Affiliations:** ^1^ National Institute of Public Health University of Southern Denmark Copenhagen Denmark; ^2^ Steno Diabetes Center Sjaelland Holbæk Denmark; ^3^ PROgrez Næstved, Slagelse and Ringsted Hospitals Slagelse Denmark; ^4^ Department of Endocrinology Nykøbing Falster Hospital, Nykøbing Falster Denmark; ^5^ Steno Diabetes Center Aarhus Aarhus University Hospital Aarhus Denmark; ^6^ Centre for General Practice, The Research Unit for General Practice in Region Zealand and Copenhagen, Section for General Practice, Department of Public Health University of Copenhagen Copenhagen Denmark

**Keywords:** cohort study, epidemiology, intellectual disability, type 2‐diabetes

## Abstract

**Background:**

Previous research shows that obesity, unhealthy eating, physical inactivity and a high use of psychotropic medications are prevalent among persons with intellectual disability (ID), which might increase the risk of type 2‐diabetes (T2DM). This study aims to investigate: (1) whether persons with ID have an increased risk of T2DM compared with an age‐ and sex‐matched reference group and (2) differences in T2DM risk by sex, birth year, ID inclusion diagnosis and ID severity.

**Methods:**

This study is a nationwide cohort study, including 65 293 persons with ID and 659 723 persons in an age‐ and sex‐matched reference group without ID. Incidence rates for T2DM were calculated and Cox proportional regression models were used to estimate adjusted hazard ratios (aHRs) for the association between ID and T2DM. Follow‐up began from the 1 January 1977 (when T2DM data were available), participants' 22nd birthday or from the date the participants immigrated to Denmark, whichever came last and continued until the onset of T2DM, emigration, death or end of follow‐up (31 December 2021), whichever came first.

**Results:**

Persons with ID had more than double risk of T2DM compared with the reference group [aHR = 2.15, 95% confidence interval (CI): 2.09–2.20]. The strongest associations were found among women, persons born between 1980 and 1999 and among persons with mild ID.

**Conclusions:**

Persons with ID have an increased risk of T2DM. This knowledge is important in relation to the development and prioritising of preventive initiatives among persons with ID in the healthcare sector. Future research should focus on the underlying mechanisms that can explain the possible association between ID and T2DM as it allows a more targeted prevention strategy.

## Introduction

Intellectual disability (ID) occurs in about 1%–2% of the general population (Maulik *et al*. 2011; Anderson *et al*. 2019). In recent years, the prevalence of ID has increased (Cooper *et al*. 2004; Bourke *et al*. 2016) as this population live longer today than previously (Thygesen *et al*. 2023). ID is a neurodevelopmental disorder characterised by marked limitations in both intellectual functioning and adaptive behaviour as expressed in conceptual, social and practical adaptive skills that originates before the age of 22 years (Schalock *et al*. 2021). It is well‐known that persons with ID experience a disproportionate burden of health inequalities compared with the general population, including poorer mental and physical health and higher mortality rates (Cooper *et al*. 2007; Tyrer *et al*. 2007; Heslop *et al*. 2014; Havercamp & Scott 2015; Thygesen *et al*. 2023). A large portion of these health disparities can be mitigated through successful health promotion, early diagnosis and improved medical care for persons living with ID (Emerson & Baines 2011). However, healthcare providers experience challenges in the care of persons with ID, partly due to lack of knowledge and skills to meet and support their needs (Powrie 2003; Williamson *et al*. 2004; Melville *et al*. 2005; Fredheim *et al*. 2013). It could also be speculated that many support personnel and staff in community‐based residential facilities without a healthcare background find it challenging to detect and respond to the health issues and illnesses of residents with ID in a timely manner. Moreover, cognitive limitations, communication difficulties and other associated conditions make it more difficult for persons with ID to access existing healthcare and prevention services and to participate in health promotion activities (Alborz *et al*. 2005; World Health Organization 2011; Ali *et al*. 2013). Knowledge about specific health problems and diseases of persons with ID remain limited as this population is underrepresented in national health surveys and clinical research (Anderson *et al*. 2019; Spaul *et al*. 2020; Bishop *et al*. 2024). Previous research has indicated that risk factors such as obesity (Hove 2004; Melville *et al*. 2007; Lloyd *et al*. 2012; Emerson *et al*. 2016; Flygare Wallen *et al*. 2018), unhealthy eating (Robertson *et al*. 2000; Hoey *et al*. 2017; Tyrer *et al*. 2020) and physical inactivity (Messent *et al*. 1998; McGuire *et al*. 2007; Bartlo & Klein 2011; Haveman *et al*. 2011; Dairo *et al*. 2016; Melville *et al*. 2018; Tyrer *et al*. 2020) are more common among persons with ID compared with the general population and therefore may be at risk of developing non‐communicable diseases such as type 2‐diabetes (T2DM). Furthermore, psychotropic medications are frequently prescribed for persons with ID, including antipsychotics, anti‐depressants, mood stabilisers and anti‐anxiety medications (Costello *et al*. 2022). Potential health hazards linked to extended usage of these medications involve the development of endocrine, metabolic and cardiovascular disorders (de Leon *et al*. 2009; Smith *et al*. 2022). It is important to obtain knowledge about the risk of T2DM among persons with ID as it is a progressive disease associated with both microvascular complications comprising diabetic kidney disease, retinopathy and neuropathy and macrovascular complications including cardiovascular diseases (Lip & Varughese 2005; Gregg *et al*. 2016; Forbes & Fotheringham 2017). Three systematic reviews have suggested a prevalence of diabetes (including both type 1 and 2) in persons with ID between 0.4% and 25% and conclude that further research is needed to obtain more accurate diabetes prevalences in this population group (McVilly *et al*. 2014; MacRae *et al*. 2015; Vancampfort *et al*. 2022). Only one systematic review has focused on T2DM alone, suggesting a prevalence between 2% and 13% (Chalk *et al*. 2016). However, whether persons with ID have an increased risk of developing T2DM compared with the general population has sparsely been investigated. A meta‐analysis comparing four prevalence studies has estimated that persons with ID have 2.46 [95% confidence interval (CI): 1.89–3.21] times higher odds for diabetes compared with the general population (Vancampfort *et al*. 2022). Only one of the included studies was able to differentiate between diabetes type suggesting that women with ID have 1.67 (95% CI: 1.39–2.01) times higher odds of T2DM compared with women without ID. For men, no association was found between ID and T2DM (Axmon *et al*. 2017). A study with high‐quality diabetes data, capable of distinguishing between diabetes types, is essential to accurately assess the risk of T2DM among persons with ID compared with the general population and to document and raise awareness of potential health inequalities. Furthermore, a comprehensive understanding of the disparities of the risk of T2DM across the severity of ID would enable better and more tailored prevention and treatment strategies for this vulnerable group in the healthcare sector. Yet no previous study has investigated the T2DM risk across the severity of ID. Hence, the overall aim of this study is to investigate whether a nationwide cohort of persons with ID have an increased risk of T2DM compared with an age‐ and sex‐matched reference group without ID. A second aim is to investigate differences in T2DM incidence by sex, birth year, ID inclusion diagnosis and ID severity.

## Methods

This study is designed as a nationwide matched cohort study. Each person with ID was matched on date of birth (±1 day) and sex with 10 persons without ID (reference group). The matching process occurred before the application of our exclusion criteria (see Persons with intellectual disability section). This resulted in a reference group that was slightly larger than what would be expected from a strict 1:10 ratio. All Danish residents have a unique personal identification number (CPR number), which was used to secure exact linkage between several high‐quality nationwide registers (Thygesen *et al*. 2011).

### Persons with intellectual disability

We included a cohort of 79 773 persons with ID in the period between 1 January 1950 and 31 December 2021 via six different nationwide Danish registers. A comprehensive description of the registers and the establishment of the cohort is described elsewhere (Thygesen *et al*. 2023). In brief, from the Danish National Patient Register (DNPR) (1977–2021) (Lynge *et al*. 2011), the Danish Psychiatric Central Research Register (1969–1994) (Mors *et al*. 2011), the Cause of Death Register (1970–2019) (Helweg‐Larsen 2011), the Danish Agency for Labour Market and Recruitment (1998–2021) and the National Cerebral Palsy Register (1950–2007) (Uldall *et al*. 2001), we included persons diagnosed with any diagnosis of ID (ICD‐8 codes; 310–315 or ICD‐10 codes; F70–F79) or a diagnosis most likely leading to ID, including Down syndrome, cerebral palsy with ID, selected congenital metabolic disorders, congenital malformations and chromosomal disorders (Table S1). From the Central Personal Registry (1968–2020) (Pedersen 2011), we identified persons with residence at institutions for persons with ID. We excluded persons who (1) had not reached the age of 22 at end of follow‐up (31 December 2021) (*n* = 11 955), (2) were diagnosed with T2DM before the age of 22 (*n* = 161), (3) died before the age of 22 or before 1 January 1977 (*n* = 1825) or (4) were not living in Denmark on their 22nd birthday or on 1 January 1977 (*n* = 539). A total of 65 293 persons was eligible for analyses (82% of the original cohort).

### Type 2‐diabetes

In Denmark, the majority (80%) of patients with T2DM are treated solely in general practice, while the remaining (20%) in particular patients with severe diabetic complications receive diabetes care in hospital outpatient clinics (Carstensen *et al*. 2020). To identify persons with T2DM treated both within the hospital sector and general practice, we used an algorithm developed by Carstensen *et al*. (Carstensen *et al*. 2020) The algorithm combines information from existing Danish health registers, including diagnoses of hospital‐treated T2DM in the DNPR (1977–2019) (Lynge *et al*. 2011), purchases of any anti‐diabetic drug (A10A; insulins or A10B; oral antidiabetics) in the Danish National Prescription Registry (1995–2021) (Kildemoes *et al*. 2011), use of diabetes podiatry in the Danish National Health Service Register (1990–2020) (Andersen *et al*. 2011) and T2DM diagnoses in the Danish Adult Diabetes Database (2005–2021) (Jorgensen *et al*. 2016). The specific criteria that must be met to be classified as a patient with T2DM are described in detail elsewhere (Carstensen *et al*. 2020). Persons with T2DM were identified in the period between 1 January 1977 and 31 December 2021.

### Statistical analysis

#### Main analyses

Descriptive characteristics were presented as frequencies with percentages, means with standard deviations (*SD*s) or medians with inter‐quartile ranges (IQRs). Furthermore, incidence rates (IRs) for T2DM were calculated and Cox proportional hazards regression models were performed to estimate the hazard ratio (HR) with 95% CIs for the association between ID and T2DM. The proportional hazards assumption was verified by visual inspection of log‐minus‐log plots. Age was used as the underlying time scale and the population was followed from the 1 January 1977 (when T2DM data was available), their 22nd birthday or from the date they immigrated to Denmark, whichever came last. We termed this date as the index date. The 22nd birthday was chosen as start time in the analyses as ID, per definition, occur before the age of 22 years. Moreover, only few people develop T2DM before the age of 22 years. The population was followed until the onset of T2DM, emigration, death or end of follow‐up (31 December 2021), whichever came first. Five Cox proportional hazards regression models were performed: (1) a main model adjusted for sex, year of birth and country of origin; (2) a model stratified on sex and adjusted for year of birth and country of origin; (3) a model stratified on year of birth adjusted for sex and country of origin; (4) a model dividing persons with ID into groups based on their ID inclusion diagnosis adjusted for sex, year of birth and country of origin; and (5) a model including persons with an ID diagnosis only, dividing this population into groups according to the severity of the ID based on the ICD‐10 diagnoses F70–F79 (profound, severe, moderate, mild and unknown). This model was adjusted for sex, year of birth and country of origin. Finally, we created a Kaplan–Meier plot to illustrate the probability of T2DM at different ages among persons with ID vs. the reference group. Data management and statistical analyses were conducted with STATA version 18.0 (StataCorp, College Station, TX, USA).

#### Sensitivity analysis

Because information on anti‐diabetic medication from the Danish National Prescription Registry and outpatient diagnoses from the DNPR is only available from 1995 and onwards, we might underestimate the incidence of T2DM cases prior to 1995. Therefore, as a sensitivity analysis, we restricted model 1 to only include T2DM cases after 1995.

### Participants

This study included 65 293 persons with ID and 659 723 persons in the reference group. There were slightly more men (53.8%) in both groups. A larger proportion of persons with ID were from Denmark compared with persons in the reference group (92.0% vs. 81.6%). Among persons with ID, 83.9% had an ID diagnosis, 2.6% had cerebral palsy, 6.7% had Down syndrome, 1.4% had metabolic disorders, 1.0% had congenital malformations/chromosomal disorders and 24.1% were identified using data regarding residence in institutions for persons with ID. These percentages sum up to more than 100% as persons with ID may have been identified through more than one data source. Persons with ID and the reference group were on average the same age at index date (approximately 29 years) and at end of follow‐up (approximately 50 years) (Table 1).

**Table 1:** Descriptive characteristics of persons with ID and the reference group

	Persons with ID (*n* = 65 293)		Reference group (*n* = 659 723)	
Sex (*n*, %)				
Male	35 121	(53.8)	354 869	(53.8)
Female	30 172	(46.2)	304 854	(46.2)
Year of birth (*n*, %)				
<1900–1919	3879	(5.9)	39 843	(6.0)
1920–1939	8512	(13.0)	84 640	(12.8)
1940–1959	13 685	(21.0)	136 669	(20.7)
1960–1979	15 918	(24.4)	164 130	(24.9)
1980–1999	23 299	(35.7)	234 441	(35.5)
Country of origin (*n*, %)				
Denmark	60 069	(92.0)	538 431	(81.6)
Immigrant or descendant from a Western country	847	(1.3)	54 581	(8.3)
Immigrant or descendant from a non‐Western country	4270	(6.5)	58 587	(8.9)
Missing	107	(0.2)	8124	(1.2)
Age at index date^†^				
Mean (*SD*)	29.1 (12.8)		29.8 (12.9)	
Median (IQR)	22 (22–31)		22 (22–33)	
Age at end of follow‐up				
Mean (*SD*)	49.2 (18.4)		51.6 (21.0)	
Median (IQR)	48 (32–63)		50 (32–69)	
Data sources (*n*, %)				
National Patient Register (somatic)	27 477	(42.1)		
National Patient Register (psychiatric)	21 939	(33.6)		
Psychiatric Central Research Register	9971	(15.3)		
Danish Register of Causes of Death	5445	(8.3)		
National Cerebral Palsy Register	1707	(2.6)		
Danish Agency of Labour Market Register	13 816	(21.2)		
Addresses at central institutions	7688	(11.8)		
Addresses at sheltered residences (2014 and 2020)	8017	(12.3)		
Inclusion diagnosis (without hierarchy) (*n*, %)^‡^				
ID diagnosis (overall)	54 801	(83.9)		
Profound intellectual disability	1659	(2.5)		
Severe intellectual disability	4050	(6.2)		
Moderate intellectual disability	7662	(11.7)		
Mild intellectual disability	26 806	(41.1)		
Unknown/other intellectual disability	31 910	(48.9)		
Cerebral palsy with intellectual disability	1707	(2.6)		
Down syndrome	4359	(6.7)		
Metabolic disorders	887	(1.4)		
Congenital malformations/chromosomal disorders	645	(1.0)		
Unknown diagnosis, from central institution	7688	(11.8)		
Unknown diagnosis, from sheltered residence (2014 and 2020)	8017	(12.3)		
Inclusion diagnosis (with hierarchy) (*n*, %)^‡^				
ID diagnosis (overall)	54 801	(83.9)		
Profound intellectual disability	1659	(2.5)		
Severe intellectual disability	3481	(5.3)		
Moderate intellectual disability	6500	(10.0)		
Mild intellectual disability	23 667	(36.2)		
Unknown/other intellectual disability	19 494	(29.9)		
Cerebral palsy with intellectual disability	669	(1.0)		
Down syndrome	2345	(3.6)		
Metabolic disorders	808	(1.2)		
Congenital malformations/chromosomal disorders	347	(0.5)		
Unknown diagnosis, from central institution	3012	(4.6)		
Unknown diagnosis, from sheltered residence (2014 and 2020)	3311	(5.1)		

^†^ Index date = the 1 January 1977 (when type 2 diabetes data were available), date of 22nd birthday, or immigration date, whichever came last.

^‡^ Hierarchy = Hierarchy means that for persons with several diagnoses or inclusion sources, only the highest in the hierarchy is used.

## Results

### Intellectual disability and type 2‐diabetes

**Figure 1:**
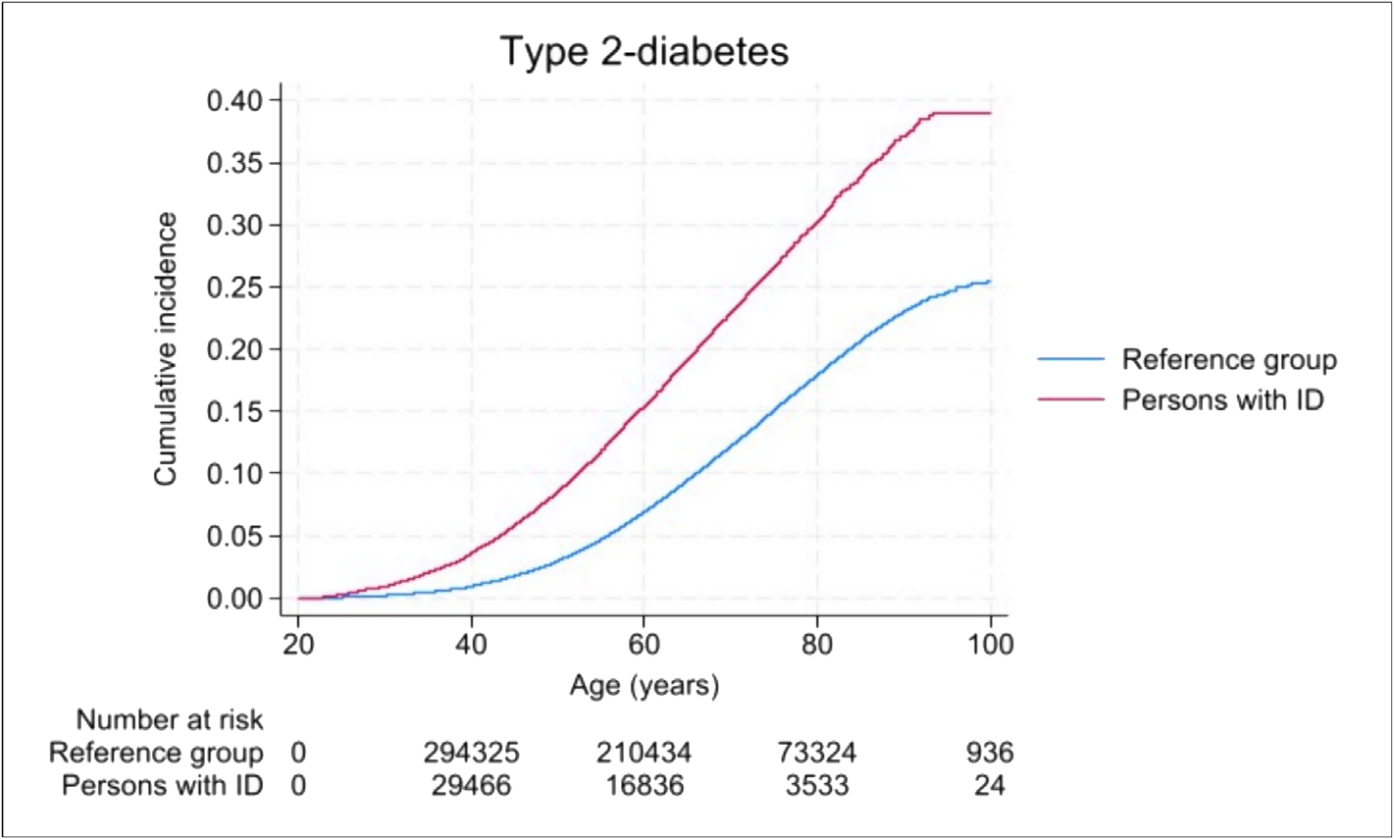
Cumulative incidence of type 2 diabetes (T2DM) among persons with intellectual disability (ID) and the reference group.

During the follow‐up period, 6249 (9.6%) persons with ID were registered with T2DM (IR = 4.8 per 1000 person‐years) compared with 39 335 (6.0%) persons in the reference group (IR = 2.7 per 1000 person‐years) (Table 2). The Kaplan–Meier plot showed a higher risk of T2DM among persons with ID compared with the reference group at all ages (Figure 1). By the age of 40 years, 3.5% had T2DM among persons with ID compared with 0.9% among persons in the reference group. Similarly, 15.4% had T2DM among persons with ID compared with 6.8% in the reference group by the age of 60 years. Table 2 presents HRs for the association between ID and T2DM. Overall, persons with ID had two times higher risk of T2DM compared with the reference group [adjusted HR (aHR) = 2.15, 95% CI: 2.09–2.20]. A stronger association was found among women (aHR = 2.63, 95% CI: 2.53–2.73) than among men (aHR = 1.78, 95% CI: 1.72–1.85). The HRs of T2DM increased with birth year, going from 1.7 times higher risk among persons with ID born between 1900–1919 to 4.8 times higher risk among persons with ID born between 1980–1999 compared with the reference group (Table 2).

**Table 2:** Incidence rates and hazard ratios for associations between ID and T2DM, stratified by sex and year of birth

	Follow‐up (Person‐years)	T2DM (*n*, %)	Rate^†^ (95% CI)	aHR (95% CI)
Model 1^‡^				
Reference group	14 339 925	39 335 (6.0)	2.74 (2.72–2.77)	1.00
Persons with ID	1 312 543	6249 (9.6)	4.76 (4.64–4.88)	2.15 (2.09–2.20)
Model 2^§^				
Women				
Reference group	7 074 975	17 045 (5.6)	2.41 (2.37–2.45)	1.00
Persons with ID	632 194	3253 (10.8)	5.15 (4.97–5.33)	2.63 (2.53–2.73)
Men				
Reference group	7 264 940	22 290 (6.3)	3.07 (3.03–3.11)	1.00
Persons with ID	680 348	2996 (8.5)	4.40 (4.23–4.56)	1.78 (1.72–1.85)
Model 3^¶^				
<1900–1919				
Reference group	696 268	2375 (6.0)	3.41 (3.28–3.55)	1.00
Persons with ID	50 284	258 (6.7)	5.13 (4.54–5.80)	1.72 (1.51–1.95)
1920–1939				
Reference group	2 645 193	11 191 (13.2)	4.23 (4.15–4.31)	1.00
Persons with ID	197 469	1286 (15.1)	6.51 (6.17–6.88)	1.88 (1.77–1.99)
1940–1959				
Reference group	5 226 764	17 503 (12.8)	3.35 (3.30–3.40)	1.00
Persons with ID	455 122	2414 (17.6)	5.30 (5.10–5.52)	1.86 (1.78–1.94)
1960–1979				
Reference group	4 138 943	7507 (4.6)	1.81 (1.77–1.86)	1.00
Persons with ID	415 821	1857 (11.7)	4.47 (4.27–4.67)	2.70 (2.57–2.84)
1980–1999				
Reference group	1 632 756	759 (0.3)	0.46 (0.43–0.50)	1.00
Persons with ID	193 847	434 (1.9)	2.24 (2.04–2.46)	4.82 (4.28–5.43)

CI, confidence interval; ID, intellectual disability; T2DM, type 2 diabetes.

^†^ Incidence per 1000 persons.

^‡^ Adjusted for sex, year of birth and country of origin.

^§^ Adjusted for year of birth and country of origin.

^¶^ Adjusted for sex and country of origin.

### Risk of type 2‐diabetes by intellectual disability inclusion diagnosis

The highest risk of T2DM was found among persons with a metabolic disorder (aHR = 5.86, 95% CI: 5.18–6.63), followed by persons with an ID diagnosis (aHR = 2.17, 95% CI: 2.11–2.24) and persons from sheltered residence (aHR = 1.74, 95% CI: 1.60–1.90) (Table 3). No associations were found among persons with cerebral palsy, Down syndrome, congenital malformations/chromosomal disorders or from central institutions (Table 3).

**Table 3:** Hazard ratios for T2DM among persons with ID compared with an age‐ and sex‐matched reference group, stratified by inclusion diagnosis

	T2DM (*n*, %)	aHR (95% CI)
Model 4^†^ ^,^ ^‡^		
ID diagnosis	5257 (9.6)	2.17 (2.11–2.24)
Cerebral palsy with intellectual disability	58 (3.4)	0.96 (0.73–1.25)
Down syndrome	150 (3.4)	0.89 (0.75–1.05)
Metabolic disorders	367 (41.4)	5.86 (5.18–6.63)
Congenital malformations/chromosomal disorders	24 (3.7)	1.04 (0.68–1.58)
Unknown diagnosis, from central institution	445 (5.9)	1.01 (0.92–1.11)
Unknown diagnosis, from sheltered residence	591 (7.4)	1.74 (1.60–1.90)

CI, confidence interval; ID, intellectual disability; T2DM, type 2 diabetes.

^†^ Adjusted for sex, year of birth and country of origin.

^‡^ Persons with ID can appear in more than one category, as they could be identified with more than one ID inclusion diagnosis.

### Risk of type 2‐diabetes by intellectual disability severity

Among persons with an ID diagnosis, the risk of T2DM decreased with the severity of ID diagnosis, from 2.6 times higher risk among persons with mild ID (aHR = 2.55, 95% CI: 2.45–2.66) to 26% lower risk among persons with profound ID (aHR = 0.74, 95% CI: 0.58–0.95) compared with the reference group (Table 4).

**Table 4:** Hazard ratios for T2DM among persons with ID compared with an age‐ and sex‐matched reference group, stratified by the severity of ID

	T2DM (*n*, %)	aHR (95% CI)
Model 5^†^ ^,^ ^‡^		
Profound intellectual disability	66 (4.0)	0.74 (0.58–0.95)
Severe intellectual disability	207 (5.9)	1.10 (0.95–1.27)
Moderate intellectual disability	510 (7.9)	1.61 (1.46–1.76)
Mild intellectual disability	2742 (11.6)	2.55 (2.45–2.66)
Unknown/other intellectual disability	1732 (8.9)	2.32 (2.21–2.45)

CI, confidence interval; ID, intellectual disability; T2DM, type 2 diabetes.

^†^ Adjusted for sex, year of birth and country of origin.

^‡^ Categorised hierarchically. Persons only appear in the group with highest level of ID severity with which they have been recorded in data sources.

### Sensitivity analysis

In the sensitivity analysis, we restricted the analysis to only include T2DM cases after 1995. This restriction did not change the main result markedly (aHR = 2.10, 95% CI: 2.04–2.16) (Table S2).

## Discussion

This study investigated the risk of T2DM among 65 293 persons with ID compared with 659 723 persons in an age‐ and sex‐matched reference group. We found that persons with ID had more than double risk of T2DM compared with the reference group. The association was stronger among women compared with men. Disparities in T2DM incidence were largest when comparing the youngest birth cohorts (1980–1999). In addition, we found a clear stepwise decrease in T2DM with increased severity of ID with highest risk among persons with mild ID.

The main findings of this study are in line with previous studies, all reporting a higher diabetes prevalence among persons with ID compared with the general population (Axmon *et al*. 2017; McCarron *et al*. 2017; Flygare Wallen *et al*. 2018; Cuypers *et al*. 2021). A Swedish register‐based cohort study by Axmon *et al*. reported that women with ID had a higher prevalence of T2DM diagnoses (5% vs. 9%) and were more often prescribed with diabetes medication (8% vs. 13%) compared with women in the general population (Axmon *et al*. 2017). No associations were found among men (Axmon *et al*. 2017). An Irish cross‐sectional study by Carron *et al*. found a diabetes prevalence of 11.1% among persons with ID compared with 6.5% among a reference group (56). Similarly, a Swedish population‐based cohort study by Wallén *et al*. found that persons with ID were 2.4 times more likely to have a recorded diagnosis of diabetes than the general population (Flygare Wallen *et al*. 2018). Lastly, a Dutch cross‐sectional population‐based study by Cuypers *et al*. showed that diabetes were 1.5 times more prevalent among persons with ID compared with people without ID (Cuypers *et al*. 2021).

Persons with ID may be at an increased risk of developing T2DM, in part, because of their limitations in intellectual functioning and adaptive behaviour, which implies that they often have difficulties in making healthy choices and access healthcare and prevention services. A high use of psychotropic medications among persons with ID has also been linked with metabolic adverse effects, including weight gain, glucose dysregulation and dyslipidaemia, which increases the risk of developing T2DM (Smith *et al*. 2022). However, further research is warranted to understand the mechanisms underlying the association between ID and T2DM.

Our finding that women with ID had a higher incidence of T2DM compared with men with ID is in line with previous research (Axmon *et al*. 2017; Flygare Wallen *et al*. 2018; Cuypers *et al*. 2021). The mechanisms behind these sex differences remain unknown. However, some research indicate that obesity, an important risk factor for T2DM, is higher among women with ID compared with men with ID (Melville *et al*. 2007). To our knowledge, this study is the first to investigate T2DM risk by the severity of ID. Persons with mild to moderate ID had a higher risk of T2DM compared with the reference group while persons with profound ID had a lower risk of T2DM compared with the reference group. These results might be explained by the fact that the prevalence of obesity is higher among adults with mild to moderate ID (27%–53%), compared with adults with severe to profound ID (11%–29%) (Cuypers *et al*. 2021). Previous research has also shown that persons with severe to profound ID are at greater risk of being underweight (Hsieh *et al*. 2014) compared with persons with mild or moderate ID due to more feeding, chewing and swallowing problems (Humphries *et al*. 2009). It has also been documented that persons with ID who live at home or in a not professionally supervised home are more likely to be obese than those who live in more supervised settings such as community‐based residential facilities (Rimmer & Yamaki 2006). In our study, it could be speculated that the increased risk of T2DM among persons with mild ID could be an expression of this group living more independently without a supportive environment, resulting in an unhealthy food intake and lack of incentive to be physically active. Future research should therefore compare the risk of T2DM in persons with ID residing in different living arrangements.

Our results show that differences in diabetes incidence between persons with ID and the reference group are largest in the youngest birth cohorts. High T2DM incidence rates among the youngest cohort of persons with ID could be explained by several factors. First, persons with ID may be exposed to risk factors such as obesity at an earlier age than the general population (Melville *et al*. 2007), which may lead to an earlier age of onset for T2DM. Another factor could be the higher frequency of GP consultations among persons with ID compared with the general population (Straetmans *et al*. 2007; Carey *et al*. 2016; Tyrer *et al*. 2024), which provides more opportunities for blood glucose testing. A third factor could be that many adolescents are prescribed psychotropic medications that require monitoring of blood glucose levels to manage potential metabolic side effects (Teeluckdharry *et al*. 2013). This increased surveillance could also contribute to the earlier diagnosis of T2DM.

Our study has strengths as well as limitations. A strength in our study is that persons with ID were identified through several data sources as it increases the completeness of the cohort. Most persons with ID (84%) were identified with a diagnosis of ID from the DNPR, which is a strength as there is a bigger insecurity about the precision of ID for those identified through institutions and disability pensions without an ID diagnosis. Generally, the quality and completeness of the DNPR is considered high (Schmidt *et al*. 2015). From the DNPR, we also had information on the severity of ID (profound, severe, moderate, mild or unknown/other), which is a strength as no previous studies has investigated the T2DM risk across the severity of ID. This information is important as our results show that individuals with mild to moderate ID might benefit from more intensive monitoring and support. A limitation in our study is that there might be some persons with ID that we cannot detect in the registers, for example those with mild ID who do not reside at institutions or have any contact to the hospitals. Persons with mild ID may therefore be slightly underrepresented in our study. Furthermore, we only included persons with ID who have survived at least until the age of 22 years. We found a higher proportion of persons with profound ID among those who died before the age of 22 years compared with those with mild ID (1.4% vs. 0.5%) (data not shown). These reflect that some children with profound ID may have long‐term and unavoidable chronic conditions, which predispose them to an early death. Persons with profound ID may therefore also be underrepresented in our cohort. A major strength in our study was the use of a comprehensive diabetes algorithm combining high‐quality data from national registers. This algorithm made it possible to distinguish between diabetes types and identify persons with T2DM treated both within the hospital sector and general practice. The multiple data sources and an average follow‐up time of 22 years increase sensitivity and data completeness. However, some research indicates that persons with ID might find it difficult to access healthcare services and have problems expressing if they are feeling sick. Therefore, persons with ID may not receive antidiabetic treatment or seek regular eye examination or podiatry to the same extent as persons without ID. If this is the case, we will detect fewer T2DM cases in the registers among persons with ID compared with persons without ID and the association between ID and T2DM might be underestimated. On the other hand, more GP consultations among persons with ID compared with the general population may increase the likelihood of blood glucose testing and thus getting a T2DM diagnosis. If this is the case, we might have overestimated the association between ID and T2DM. A potential weakness of our study is the inability to explore potential mediators in the association between ID and T2DM. These include obesity, unhealthy eating patterns, physical inactivity and use of psychotropic medication. Future research investigating these factors as potential mediators is important for the understanding of causal pathways between ID and T2DM and also for developing effective and targeted interventions. However, such analyses were beyond the scope of this study.

In conclusion, persons with ID have an increased risk of T2DM compared with an age‐ and sex‐matched reference group without ID. This knowledge is important in relation to the development and prioritising of preventive initiatives in the healthcare sector. Our results suggest that initiatives should focus on women with ID and those with mild to moderate ID where incidence of T2DM appears to be highest. However, based on our knowledge about general problems with undiagnosed health conditions and difficulties with timely recognising health symptoms in persons with ID, we believe that there is a need for broader strategies that improve the recognition, diagnosis and management of T2DM across the entire ID population. This study could argue that a different strategy in general for chronic diseases among people with ID is needed, not only for T2DM but also for other chronic diseases like chronic obstructive pulmonary disease and cardiovascular diseases, which are also abundant in this group, thereby improving treatment results and better preventive measures. Future research should also focus on the underlying mechanisms that can explain the possible association between ID and T2DM as it allows a more targeted prevention strategy.

## Conflict of Interest

None.

## Source of Funding

The support for this study was provided by the National Institute of Public Health, University of Southern Denmark and Steno Diabetes Center Sjaelland.

## Ethics Approval Statement

This study was approved by the University of Southern Denmark (no. 11.215).

## Supporting information


**Table S1.** Diagnoses used to identify persons with intellectual disabilities (ID).
**Table S2**. Overall rates of T2DM among persons with ID and the reference group, included only T2DM cases after 1995 (*N* = 679 131).

## Data Availability

The data used in this study are available by application from the Danish National Board of Health, Statistics Denmark and from the clinical quality databases (RKKP).

## References

[jir13190-bib-0001] Alborz A. , McNally R. & Glendinning C. (2005) Access to health care for people with learning disabilities in the UK: mapping the issues and reviewing the evidence. Journal of Health Services Research & Policy 10, 173–182. 16053595 10.1258/1355819054338997 PMC2020839

[jir13190-bib-0002] Ali A. , Scior K. , Ratti V. , Strydom A. , King M. & Hassiotis A. (2013) Discrimination and other barriers to accessing health care: perspectives of patients with mild and moderate intellectual disability and their carers. PLoS ONE 8, e70855. 23951026 10.1371/journal.pone.0070855 PMC3741324

[jir13190-bib-0003] Andersen J. S. , Olivarius Nde F. & Krasnik A. (2011) The Danish National Health Service Register. Scandinavian Journal of Public Health 39, 34–37. 21775348 10.1177/1403494810394718

[jir13190-bib-0004] Anderson L. L. , Larson S. A. , MapelLentz S. & Hall‐Lande J. (2019) A systematic review of U.S. studies on the prevalence of intellectual or developmental disabilities since 2000. Intellectual and Developmental Disabilities 57, 421–438. 31568738 10.1352/1934-9556-57.5.421

[jir13190-bib-0005] Axmon A. , Ahlstrom G. & Hoglund P. (2017) Prevalence and treatment of diabetes mellitus and hypertension among older adults with intellectual disability in comparison with the general population. BMC Geriatrics 17, 272. 29169334 10.1186/s12877-017-0658-2 PMC5701367

[jir13190-bib-0006] Bartlo P. & Klein P. J. (2011) Physical activity benefits and needs in adults with intellectual disabilities: systematic review of the literature. American Journal on Intellectual and Developmental Disabilities 116, 220–232. 21591845 10.1352/1944-7558-116.3.220

[jir13190-bib-0007] Bishop R. , Laugharne R. , Shaw N. , Russell A. M. , Goodley D. , Banerjee S. *et al*. (2024) The inclusion of adults with intellectual disabilities in health research—challenges, barriers and opportunities: a mixed‐method study among stakeholders in England. Journal of Intellectual Disability Research 68, 140–149. 37815212 10.1111/jir.13097

[jir13190-bib-0008] Bourke J. , de Klerk N. , Smith T. & Leonard H. (2016) Population‐based prevalence of intellectual disability and autism spectrum disorders in western australia: a comparison with previous estimates. Medicine 95, e3737. 27227936 10.1097/MD.0000000000003737 PMC4902360

[jir13190-bib-0009] Carey I. M. , Shah S. M. , Hosking F. J. , DeWilde S. , Harris T. , Beighton C. *et al*. (2016) Health characteristics and consultation patterns of people with intellectual disability: a cross‐sectional database study in English general practice. The British Journal of General Practice 66, e264–e270. 26906630 10.3399/bjgp16X684301 PMC4809710

[jir13190-bib-0010] Carstensen B. , Ronn P. F. & Jorgensen M. E. (2020) Prevalence, incidence and mortality of type 1 and type 2 diabetes in Denmark 1996–2016. BMJ Open Diabetes Research & Care 8, e001071. 10.1136/bmjdrc-2019-001071 PMC7265004 32475839

[jir13190-bib-0011] Chalk T. , Dunkley A. , Gray L. , Spong R. , Gangadharan S. , Davies M. *et al*. (2016) Rates of type 2 diabetes, cardiovascular disease and associated risk factors in people with intellectual disability populations: Systematic review and meta‐analysis. Diabetic Medicine 33, 73–74.

[jir13190-bib-0012] Cooper S. A. , Melville C. & Morrison J. (2004) People with intellectual disabilities. BMJ 329, 414–415. 15321883 10.1136/bmj.329.7463.414 PMC514194

[jir13190-bib-0013] Cooper S. A. , Smiley E. , Morrison J. , Williamson A. & Allan L. (2007) Mental ill‐health in adults with intellectual disabilities: prevalence and associated factors. The British Journal of Psychiatry 190, 27–35. 17197653 10.1192/bjp.bp.106.022483

[jir13190-bib-0014] Costello A. , Hudson E. , Morrissey S. , Sharma D. , Kelly D. & Doody O. (2022) Management of psychotropic medications in adults with intellectual disability: a scoping review. Annals of Medicine 54, 2486–2499. 36120887 10.1080/07853890.2022.2121853 PMC9518601

[jir13190-bib-0015] Cuypers M. , Leijssen M. , Bakker‐van Gijssel E. J. , Pouls K. P. M. , Mastebroek M. M. , Naaldenberg J. *et al*. (2021) Patterns in the prevalence of diabetes and incidence of diabetic complications in people with and without an intellectual disability in Dutch primary care: insights from a population‐based data‐linkage study. Primary Care Diabetes 15, 372–377. 33323353 10.1016/j.pcd.2020.11.012

[jir13190-bib-0016] Dairo Y. M. , Collett J. , Dawes H. & Oskrochi G. R. (2016) Physical activity levels in adults with intellectual disabilities: a systematic review. Preventive Medical Reports 4, 209–219. 10.1016/j.pmedr.2016.06.008 PMC4929079 27413684

[jir13190-bib-0017] de Leon J. , Greenlee B. , Barber J. , Sabaawi M. & Singh N. N. (2009) Practical guidelines for the use of new generation antipsychotic drugs (except clozapine) in adult individuals with intellectual disabilities. Research in Developmental Disabilities 30, 613–669. 19084370 10.1016/j.ridd.2008.10.010

[jir13190-bib-0018] Emerson E. & Baines S. (2011) Health inequalities and people with learning disabilities in the UK. Tizard Learning Disability Review. 16, 42–48.

[jir13190-bib-0019] Emerson E. , Robertson J. , Baines S. & Hatton C. (2016) Obesity in British children with and without intellectual disability: cohort study. BMC Public Health 16, 644. 27460572 10.1186/s12889-016-3309-1 PMC4962444

[jir13190-bib-0020] Flygare Wallen E. , Ljunggren G. , Carlsson A. C. , Pettersson D. & Wandell P. (2018) High prevalence of diabetes mellitus, hypertension and obesity among persons with a recorded diagnosis of intellectual disability or autism spectrum disorder. Journal of Intellectual Disability Research 62, 269–280. 29280230 10.1111/jir.12462

[jir13190-bib-0021] Forbes J. M. & Fotheringham A. K. (2017) Vascular complications in diabetes: old messages, new thoughts. Diabetologia 60, 2129–2138. 28725914 10.1007/s00125-017-4360-x

[jir13190-bib-0022] Fredheim T. , Haavet O. R. , Danbolt L. J. , Kjonsberg K. & Lien L. (2013) Intellectual disability and mental health problems: a qualitative study of general practitioners' views. BMJ Open 3, e002283. 10.1136/bmjopen-2012-002283 PMC3612780 23471607

[jir13190-bib-0023] Gregg E. W. , Sattar N. & Ali M. K. (2016) The changing face of diabetes complications. The Lancet Diabetes and Endocrinology 4, 537–547. 27156051 10.1016/S2213-8587(16)30010-9

[jir13190-bib-0024] Haveman M. , Perry J. , Salvador‐Carulla L. , Walsh P. N. , Kerr M. , Van Schrojenstein Lantman‐de Valk H. *et al*. (2011) Ageing and health status in adults with intellectual disabilities: results of the European POMONA II study. Journal of Intellectual & Developmental Disability 36, 49–60. 21314593 10.3109/13668250.2010.549464

[jir13190-bib-0025] Havercamp S. M. & Scott H. M. (2015) National health surveillance of adults with disabilities, adults with intellectual and developmental disabilities, and adults with no disabilities. Disability and Health Journal 8, 165–172. 25595297 10.1016/j.dhjo.2014.11.002

[jir13190-bib-0026] Helweg‐Larsen K. (2011) The Danish Register of Causes of Death. Scandinavian Journal of Public Health 39, 26–29. 10.1177/1403494811399958 21775346

[jir13190-bib-0027] Heslop P. , Blair P. S. , Fleming P. , Hoghton M. , Marriott A. & Russ L. (2014) The confidential inquiry into premature deaths of people with intellectual disabilities in the UK: a population‐based study. Lancet 383, 889–895. 24332307 10.1016/S0140-6736(13)62026-7

[jir13190-bib-0028] Hoey E. , Staines A. , Walsh D. , Corby D. , Bowers K. , Belton S. *et al*. (2017) An examination of the nutritional intake and anthropometric status of individuals with intellectual disabilities: results from the SOPHIE study. Journal of intellectual disabilities: JOID 21, 346–365. 27402617 10.1177/1744629516657946

[jir13190-bib-0029] Hove O. (2004) Weight survey on adult persons with mental retardation living in the community. Research in Developmental Disabilities 25, 9–17. 14733973 10.1016/j.ridd.2003.04.004

[jir13190-bib-0030] Hsieh K. , Rimmer J. H. & Heller T. (2014) Obesity and associated factors in adults with intellectual disability. Journal of Intellectual Disability Research 58, 851–863. 24256455 10.1111/jir.12100

[jir13190-bib-0031] Humphries K. , Traci M. A. & Seekins T. (2009) Nutrition and adults with intellectual or developmental disabilities: systematic literature review results. Intellectual and Developmental Disabilities 47, 163–185. 19489663 10.1352/1934-9556-47.3.163

[jir13190-bib-0032] Jorgensen M. E. , Kristensen J. K. , Reventlov Husted G. , Cerqueira C. & Rossing P. (2016) The Danish Adult Diabetes Registry. Clinical Epidemiology 8, 429–434. 27843339 10.2147/CLEP.S99518 PMC5098513

[jir13190-bib-0033] Kildemoes H. W. , Sorensen H. T. & Hallas J. (2011) The Danish National Prescription Registry. Scandinavian Journal of Public Health 39, 38–41. 21775349 10.1177/1403494810394717

[jir13190-bib-0034] Lip G. Y. & Varughese G. I. (2005) Diabetes mellitus and atrial fibrillation: perspectives on epidemiological and pathophysiological links. International Journal of Cardiology 105, 319–321. 16274776 10.1016/j.ijcard.2005.03.003

[jir13190-bib-0035] Lloyd M. , Temple V. A. & Foley J. T. (2012) International BMI comparison of children and youth with intellectual disabilities participating in Special Olympics. Research in Developmental Disabilities 33, 1708–1714. 22699244 10.1016/j.ridd.2012.04.014

[jir13190-bib-0036] Lynge E. , Sandegaard J. L. & Rebolj M. (2011) The Danish National Patient Register. Scandinavian Journal of Public Health 39, 30–33. 21775347 10.1177/1403494811401482

[jir13190-bib-0037] MacRae S. , Brown M. , Karatzias T. , Taggart L. , Truesdale‐Kennedy M. , Walley R. *et al*. (2015) Diabetes in people with intellectual disabilities: a systematic review of the literature. Research in Developmental Disabilities 47, 352–374. 26496008 10.1016/j.ridd.2015.10.003

[jir13190-bib-0038] Maulik P. K. , Mascarenhas M. N. , Mathers C. D. , Dua T. & Saxena S. (2011) Prevalence of intellectual disability: a meta‐analysis of population‐based studies. Research in Developmental Disabilities 32, 419–436. 21236634 10.1016/j.ridd.2010.12.018

[jir13190-bib-0039] McCarron M. , Cleary E. & McCallion P. (2017) Health and health‐care utilization of the older population of ireland: comparing the intellectual disability population and the general population. Research on Aging 39, 693–718. 28566009 10.1177/0164027516684172

[jir13190-bib-0040] McGuire B. E. , Daly P. & Smyth F. (2007) Lifestyle and health behaviours of adults with an intellectual disability. Journal of Intellectual Disability Research 51, 497–510. 17537163 10.1111/j.1365-2788.2006.00915.x

[jir13190-bib-0041] McVilly K. , McGillivray J. , Curtis A. , Lehmann J. , Morrish L. & Speight J. (2014) Diabetes in people with an intellectual disability: a systematic review of prevalence, incidence and impact. Diabetic Medicine 31, 897–904. 24824086 10.1111/dme.12494

[jir13190-bib-0042] Melville C. A. , Finlayson J. , Cooper S. A. , Allan L. , Robinson N. , Burns E. *et al*. (2005) Enhancing primary health care services for adults with intellectual disabilities. Journal of Intellectual Disability Research 49, 190–198. 15713194 10.1111/j.1365-2788.2005.00640.x

[jir13190-bib-0043] Melville C. A. , Hamilton S. , Hankey C. R. , Miller S. & Boyle S. (2007) The prevalence and determinants of obesity in adults with intellectual disabilities. Obesity Reviews 8, 223–230. 17444964 10.1111/j.1467-789X.2006.00296.x

[jir13190-bib-0044] Melville C. A. , McGarty A. , Harris L. , Hughes‐McCormack L. , Baltzer M. , McArthur L. A. *et al*. (2018) A population‐based, cross‐sectional study of the prevalence and correlates of sedentary behaviour of adults with intellectual disabilities. Journal of Intellectual Disability Research 62, 60–71. 29214701 10.1111/jir.12454

[jir13190-bib-0045] Messent P. R. , Cooke C. B. & Long J. (1998) Daily physical activity in adults with mild and moderate learning disabilities: is there enough? Disability and Rehabilitation 20, 424–427. 9846242 10.3109/09638289809166104

[jir13190-bib-0046] Mors O. , Perto G. P. & Mortensen P. B. (2011) The Danish Psychiatric Central Research Register. Scandinavian Journal of Public Health 39, 54–57. 21775352 10.1177/1403494810395825

[jir13190-bib-0047] Pedersen C. B. (2011) The Danish Civil Registration System. Scandinavian Journal of Public Health 39, 22–25. 21775345 10.1177/1403494810387965

[jir13190-bib-0048] Powrie E. (2003) Primary health care provision for adults with a learning disability. Journal of Advanced Nursing 42, 413–423. 12752886 10.1046/j.1365-2648.2003.02633.x

[jir13190-bib-0049] Rimmer J. H. & Yamaki K. (2006) Obesity and intellectual disability. Mental Retardation and Developmental Disabilities Research Reviews 12, 22–27. 16435329 10.1002/mrdd.20091

[jir13190-bib-0050] Robertson J. , Emerson E. , Gregory N. , Hatto C. , Turner S. , Kessissoglou S. *et al*. (2000) Lifestyle related risk factors for poor health in residential settings for people with intellectual disabilities. Research in Developmental Disabilities 21, 469–486. 11153830 10.1016/s0891-4222(00)00053-6

[jir13190-bib-0051] Schalock R. L. , Luckasson R. & Tasse M. J. (2021) An overview of intellectual disability: definition, diagnosis, classification, and systems of supports (12th ed.). American Journal on Intellectual and Developmental Disabilities 126, 439–442. 34700345 10.1352/1944-7558-126.6.439

[jir13190-bib-0052] Schmidt M. , Schmidt S. A. , Sandegaard J. L. , Ehrenstein V. , Pedersen L. & Sorensen H. T. (2015) The Danish National Patient Registry: a review of content, data quality, and research potential. Clinical Epidemiology 7, 449–490. 26604824 10.2147/CLEP.S91125 PMC4655913

[jir13190-bib-0053] Smith E. , Stogios N. , Au E. , Maksyutynska K. , De R. , Ji A. *et al*. (2022) The metabolic adverse effects of antipsychotic use in individuals with intellectual and/or developmental disability: a systematic review and meta‐analysis. Acta Psychiatrica Scandinavica 146, 201–214. 35894550 10.1111/acps.13484

[jir13190-bib-0054] Spaul S. W. , Hudson R. , Harvey C. , Macdonald H. & Perez J. (2020) Exclusion criterion: learning disability. Lancet 395, e29. 32061300 10.1016/S0140-6736(20)30051-9

[jir13190-bib-0055] Straetmans J. M. , van Schrojenstein Lantman‐de H. M. , Schellevis F. G. & Dinant G. J. (2007) Health problems of people with intellectual disabilities: the impact for general practice. The British Journal of General Practice 57, 64–66. 17244427 PMC2032703

[jir13190-bib-0056] Teeluckdharry S. , Sharma S. , O'Rourke E. , Tharian P. , Gondalekar A. , Nainar F. *et al*. (2013) Monitoring metabolic side effects of atypical antipsychotics in people with an intellectual disability. Journal of intellectual disabilities: JOID 17, 223–235. 23801356 10.1177/1744629513495261

[jir13190-bib-0057] Thygesen L. C. , Daasnes C. , Thaulow I. & Brønnum‐Hansen H. (2011) Introduction to Danish (nationwide) registers on health and social issues: structure, access, legislation, and archiving. Scandinavian Journal of Public Health 39, 12–16. 21898916 10.1177/1403494811399956

[jir13190-bib-0058] Thygesen L. C. , Lassen T. H. , Horsbøl T. A. , Mairey I. P. , Juel K. , Hoei‐Hansen C. E. *et al*. (2023) Mortality patterns in a Danish nationwide cohort of persons with intellectual disabilities. Journal of intellectual disabilities: JOID., 17446295231154102. 10.1177/17446295231154102 36723454

[jir13190-bib-0059] Tyrer F. , Ling S. , Bhaumik S. , Gangadharan S. K. , Khunti K. , Gray L. J. *et al*. (2020) Diabetes in adults with intellectual disability: prevalence and associated demographic, lifestyle, independence and health factors. Journal of Intellectual Disability Research 64, 287–295. 31976599 10.1111/jir.12718

[jir13190-bib-0060] Tyrer F. , Morriss R. , Kiani R. , Gangadharan S. K. , Kundaje H. & Rutherford M. J. (2024) Comparing the number and length of primary care consultations in people with and without intellectual disabilities and health needs: observational cohort study using electronic health records. Family Practice 41, 501–509. 36440948 10.1093/fampra/cmac135 PMC11324320

[jir13190-bib-0061] Tyrer F. , Smith L. K. & McGrother C. W. (2007) Mortality in adults with moderate to profound intellectual disability: a population‐based study. Journal of Intellectual Disability Research 51, 520–527. 17537165 10.1111/j.1365-2788.2006.00918.x

[jir13190-bib-0062] Uldall P. , Michelsen S. I. , Topp M. & Madsen M. (2001) The Danish Cerebral Palsy Registry. A registry on a specific impairment. Danish Medical Bulletin 48, 161–163. 11556266

[jir13190-bib-0063] Vancampfort D. , Schuch F. , Van Damme T. , Firth J. , Suetani S. , Stubbs B. *et al*. (2022) Prevalence of diabetes in people with intellectual disabilities and age‐ and gender‐matched controls: a meta‐analysis. Journal of Applied Research in Intellectual Disabilities 35, 301–311. 34658096 10.1111/jar.12949

[jir13190-bib-0064] Williamson A. , Allan L. , Cooper S. A. , Morrison J. & Curtice L. (2004) The general practitioner interface with people with intellectual disabilities and their supports. The European Journal of General Practice 10, 66–70. 15232527 10.3109/13814780409094236

[jir13190-bib-0065] World Health Organization . World report on disability. 2011. 26131540

