# Supplementary material for: The risk of type 2‐diabetes among persons with intellectual disability: a Danish population‐based matched cohort study
Source: J Intellect Disabil Res. 2024 Oct 2;69(1):90–102. doi: 10.1111/jir.13190 (PMC11621590; doi:10.1111/jir.13190)
Supplement: Supplementary file 1 — Table S1. Diagnoses used to identify persons with intellectual disabilities (ID). Table S2. Overall rates of T2DM among persons with ID and the reference group, included only T2DM cases after 1995 (N = 679 131). [file JIR-69-90-s001.docx]

**Supplementary Table 1.** Diagnoses used to identify persons with intellectual disabilities (ID).

| Diagnosis | ICD-8 | ICD-10 |
| --- | --- | --- |
| Mild ID | 310-311 | F70 |
| Moderate ID | 312 | F71 |
| Severe ID | 313 | F72 |
| Profound ID | 314 | F73 |
| Other ID and ID without further specification | 315 | F78, F79 |
| Cerebral palsy and ID | - | G80 (a) |
| Down syndrome | 759.3 | Q90 |
| Metabolic disorders likely to result in ID | 271.2 | E72.0E, E72.5A, E72.8E, E74.2B, E74.4B, E74.4C, E75.0, E75.1, E75.2D, E75.2E, E75.2G, E75.2H, E75.3, E75.4, E75.5A,  E75.5B, E77, E79.1 |
| Congenital malformation and chromosomal disorders likely to result in ID | 759.6 | Q85.1, Q93.5C, Q93.8A, Q99 |

(a) Persons with cerebral palsy is not identified by ICD-10 codes but through child neurologist medical record review.

**Supplementary Table 2**. Overall rates of T2DM among persons with ID and the reference group, included only T2DM cases after 1995 (N=679,131).

|  | **Follow-up**  **(Person-years)** | **T2DM (n, %)** | **Rate^a^ (95 % CI)** | **aHR^b^ (95 % CI)** |
| --- | --- | --- | --- | --- |
|  |  |  |  |  |
| Reference group | 9,642,401 | 36,503 (5.9) | 3.79 (3.75-3.82) | 1.00 |
| Persons with ID | 859,204 | 5,570 (9.6) | 6.48 (6.31-6.66) | 2.10 (2.04-2.16) |

^a^Incidence per 1,000 persons.

^b^Adjusted for sex, year of birth and country of origin.
